# Ice Cream

**Published:** 1888-09

**Authors:** 


### ICE CREAM.

As the seasen becomes more advanced and the days torrid with intense heat, we find the demand for ice cream at its highest.

A number of samples of ice cream were selected in several parts of Philadelphia, and only in one instance did we find any of the samples free from either gelatine, starch or artificial coloring. A strawberry cream was found to contain analine to color red. Gelatine, if pure, is perhaps permissable in making fancy molds of ice cream, but starch is a cheap adulterant, and analine is a dangerous coloring and should not be found in the cream under any circumstances. Analines generally contain arsenic or other poisonous chemicals to develop the colors.

However, if tumeric starch, gelatine, etc., have to be used in order to supply cheap trade, then let the people be informed that one kind of cream at a reduced price is made of cream or milk, gelatine, starch, etc., and that the other kind, at a higher price, is made of pure cream and sugar. There can be no objection to the adulterating of the cream with harmless matter and the selling of it as it is. The selling of things for what they are, from a spavined horse down to a pint of milk, is the requisite.—*Anti-Adulteration Journal.*

